# Imported Human Rabies — Kentucky and Ohio, 2024

**DOI:** 10.15585/mmwr.mm7502a3

**Published:** 2026-01-15

**Authors:** Alexandra Barger, Sara F. Margrey, Allison W. Siu, Ryan Wallace, Rebecca Earnest, Molly Frankel, Hermella Eshete, Carolyn Swisshelm, Eli Shiltz, Kimberly Wright, Arminda Allen, Lillian A. Orciari, Crystal M. Gigante, Rene Condori, Pamela Yager, Michael Niezgoda, Panayampalli S. Satheshkumar, Douglas Thoroughman, Kathleen Winter, Kelly Giesbrecht

**Affiliations:** ^1^Kentucky Department for Public Health; ^2^Division of Workforce Development, National Center for State, Tribal, Local, and Territorial Public Health Infrastructure and Workforce, CDC; ^3^Ohio Department of Health; ^4^Division of State and Local Readiness, Office of Readiness and Response, CDC; ^5^Division of High-Consequence Pathogens and Pathology, National Center for Emerging and Zoonotic Infectious Diseases, CDC; ^6^Northern Kentucky Health Department, Florence, Kentucky; ^7^Cincinnati Health Department, Cincinnati, Ohio.

**Summary**

**What is already known about this topic?**

Human rabies cases are rare in the United States; most result from domestic wildlife exposure. U.S. residents can acquire rabies abroad, typically through contact with dogs in areas where dog-maintained rabies is endemic.

**What is added by this report?**

A man who relocated to the United States from Haiti later died from infection with a dog-maintained rabies virus acquired in Haiti. Rabies diagnosis was delayed, and standard infection control precautions were not uniformly used during his medical care, leading to risk assessments of 709 contacts across three states and recommendations for postexposure prophylaxis for 60 persons, 88% of whom were health care workers.

**What are the implications for public health practice?**

Prompt diagnosis of human rabies is essential to limit potential exposure of health care workers and other contacts. Use of standard infection control precautions, recommended for all patient care, can help prevent exposure.

## Abstract

Human rabies cases are rare in the United States; most result from domestic wildlife exposure. U.S. residents can acquire rabies abroad, typically through contact with dogs in areas where dog-maintained rabies is endemic. In November 2024, a man from Haiti was admitted to a Kentucky hospital with an 8-day history of progressive lower extremity pain and weakness. Soon after admission, he experienced hypersalivation, dysphagia, agitation, and eventually, respiratory failure requiring invasive mechanical ventilation. Ten days after admission, he was transferred to a referral hospital in Ohio, where his condition further deteriorated. Despite early consideration of rabies in the differential diagnosis, testing was delayed until late in the clinical course while other diagnostic possibilities were pursued. Rabies testing was initiated on the 29th hospital day and was confirmed 5 days later; the patient died that day. Phylogenetic analysis of the nucleoprotein gene supported acquisition of a dog-maintained rabies virus variant in Haiti. In total, 709 possible contacts during the patient’s infectious period underwent risk assessment; 60 (8%) were recommended to receive rabies postexposure prophylaxis (PEP) because of exposure to saliva. Before the patient’s rabies diagnosis, standard precautions were used inconsistently during his care; among 60 persons recommended to receive PEP, 52 (88%) were health care workers. Earlier rabies diagnosis and regular adherence to standard infection control precautions, recommended for all patient care, might have reduced health care–associated exposures. This case underscores the importance of early public health consultation upon clinical suspicion of rabies and universal adherence to standard precautions.

## Introduction

In November 2024, a man from Haiti who had been living in the United States for approximately 7 months sought care in a Kentucky emergency department three times over 4 days for progressive lower extremity weakness and pain; he was hospitalized, and shortly thereafter he experienced agitation and hypersalivation. Ten days later, after further neurologic deterioration, he was transferred to a referral hospital in Ohio. Although rabies was considered early in the patient’s hospital course, in the absence of reported animal exposure, other diagnoses were initially pursued, and rabies testing was not sought for several weeks. Rabies testing was initiated on the 29th hospital day, and the diagnosis was confirmed by CDC 5 days later, the same day that the patient died. Analysis indicated that the virus was consistent with a rabies virus variant found in dogs in Haiti, one of the countries with the highest risk for rabies in the Western Hemisphere (*1*). An extensive contact tracing effort was undertaken to identify persons who might have been exposed to the patient’s infectious material and to recommend postexposure prophylaxis when indicated. This report describes the patient’s signs and symptoms, hospital course, and the subsequent contact tracing activities once a diagnosis of rabies was confirmed.

## Investigation and Results

### Clinical Signs and Symptoms and Initial Hospitalization

Information about the patient’s clinical and hospitalization course was provided by the treating facilities through the local health departments. In April 2024, the patient had relocated from Haiti to the United States; he began working in a Kentucky warehouse in August (Figure). Three months later, in November, he sought treatment at a local emergency department (hospital A) for a 4-day history of knee and lower back pain. Knee and spine radiographs were normal, and he was discharged. He returned later the same day with worsening pain in both legs, nausea, and urinary frequency. Clinicians administered intravenous fluids and pain medication and discharged him again. No specific diagnosis other than musculoskeletal pain was documented. Two days later, he returned with dizziness and severe leg weakness and required assistance walking. Computed tomography of the head was normal, but magnetic resonance imaging of the lumbar spine revealed a bulging intervertebral disc; this was interpreted as a plausible mechanism for radiculopathy and the cause of his symptoms. He initially declined hospital admission, but the following day (hospital day 1), he returned to hospital A by ambulance after losing the ability to walk, experiencing weakness that had progressed to his arms, and experiencing respiratory difficulty. He was admitted to the hospital, and clinicians initiated an extensive evaluation in consultation with neurology and infectious disease specialists. On hospital day 2, he developed hypersalivation, dysphagia, and agitation, and by hospital day 3, progressive neurologic decline necessitated endotracheal intubation and invasive mechanical ventilation.

**FIGURE:**
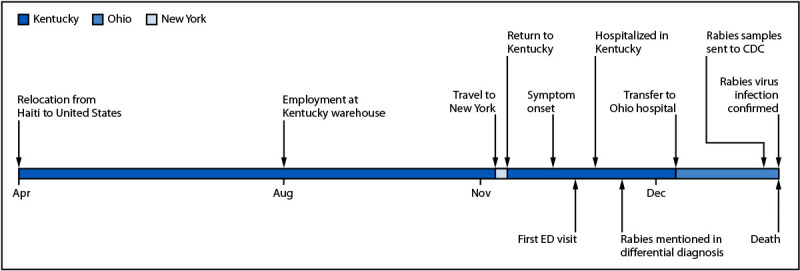
Timeline for human rabies case imported from Haiti — Kentucky and Ohio, 2024 **Abbreviation:** ED = emergency department.

### Hospital A Course

Cerebrospinal fluid (CSF) from a lumbar puncture on hospital day 3 tested positive for toxoplasma immunoglobulin G (IgG); all other tests for infectious, autoimmune, and neoplastic etiologies were negative. Clinicians considered rabies in the differential diagnosis as early as hospital day 3; however, because of the critical nature of the patient’s illness at that time, he was unable to respond to questions about animal exposure, and family members interviewed during the hospitalization were unaware of any animal exposure. Therefore, in the absence of known exposure, rabies testing was not initially pursued, in favor of plausible alternative diagnoses. The bulging lumbar disc was initially considered the likely cause of his leg weakness, but this did not explain his other symptoms. Recent receipt of several vaccines raised suspicion for Guillain-Barré syndrome, prompting treatment with intravenous immunoglobulin. He also received empiric treatment for central nervous system toxoplasmosis.

### Transfer to Hospital B and Request for Rabies Testing

On hospital day 10, the patient experienced status epilepticus, requiring increasing sedation. He was transferred to an Ohio hospital (hospital B) for neurocritical care on hospital day 13. Because of the hypersalivation, he underwent salivary gland biopsy on hospital day 16; pathologic examination found nonspecific inflammation. A brain magnetic resonance imaging study on hospital day 17 showed anoxic injury with severe ventricular effacement (i.e., obliteration of the ventricular space as a consequence of mass effect) and brain stem herniation. Computed tomography angiography showed no cerebral blood flow.

On hospital day 29, physicians at hospital B consulted the Kentucky Department for Public Health (KDPH) and the Ohio Department of Health (ODH) to request rabies testing. Serum, CSF, saliva, and nuchal skin biopsy samples were sent to CDC by the ODH laboratory. The samples were received by CDC on hospital day 34, and rabies was confirmed later that day by the detection of rabies IgG and immunoglobulin M by indirect immunofluorescence assay in serum and CSF and by detection of rabies virus RNA by real-time reverse transcription–polymerase chain reaction in one of two saliva samples (*2*,*3*). The patient died on hospital day 34, 40 days after symptom onset.

Rabies virus neutralizing antibodies were later detected in serum and CSF by the rapid fluorescent focus inhibition test. The rabies virus RNA signal in nuchal skin was below the positivity threshold and was reported as inconclusive.

### Identification of Rabies Virus Variant

Postmortem sampling of brain tissue was conducted by needle aspiration through the foramen magnum. Antigenic typing revealed a rabies virus variant similar to that found in Caribbean dogs and mongooses. Genomic sequencing and phylogenetic analysis of the complete nucleoprotein gene was consistent with rabies virus found in dogs in Haiti (Cosmopolitan clade, Haiti-Dominican Republic variant CAR1a).*

## Public Health Response

### Epidemiologic Investigation

After confirming rabies infection, KDPH, ODH, the Northern Kentucky Health Department, the Cincinnati Health Department, and CDC coordinated response activities. These activities were reviewed by CDC, deemed not research, and were conducted consistent with applicable federal law and CDC policy.^†^ The participating health agencies considered these activities to be part of routine public health practice that did not require human subjects review.

### Haiti Public Health Notification and Field Investigation

Rabies virus variant typing and sequencing results indicated that the patient had acquired rabies in Haiti, obviating the need for further U.S. animal source investigation. CDC issued a public health notification to Haiti, recommending follow-up to identify the exposure source and assess additional persons who had been exposed to the rabid animal and who might need postexposure prophylaxis (PEP). A field investigation team from Haiti’s National Animal Rabies Surveillance Program was deployed to the patient’s family’s last known location to conduct in-person interviews. Their investigation did not identify any definite animal exposures. One report that the patient might have been scratched by a cat could not be verified. The patient had also traveled extensively within Haiti, precluding ascertainment of the source of his rabies exposure.

### Contact Tracing

**Health care contacts.** Public health officials defined the infectious period as 14 days before symptom onset until the patient’s death (*4*). Exposure was defined as contact between the patient’s infectious body fluid or tissue and a contact’s mucous membrane or broken skin. KDPH and the Northern Kentucky Health Department developed an online risk assessment plan to standardize data collection. Infection prevention specialists at hospitals A and B, in consultation with public health officials, identified potentially exposed employees at their respective facilities. A standardized questionnaire was administered to 645 employees, including 451 at hospital A and 194 at hospital B (Table). To collect additional information, telephone interviews were conducted with persons who reported possible contact with tears, saliva, or neural tissue. During the interview, details of the possible exposure, including the nature of the body fluid contact and use of personal protective equipment (PPE), were discussed. If a health care worker used PPE that prevented contact between the patient’s infectious body fluid and the health care worker’s mucous membranes or broken skin, the health care worker was not considered exposed.

**TABLE:** Number of contacts of a patient with rabies, recommendations to receive rabies postexposure prophylaxis, and completion of postexposure prophylaxis, by contact group — Kentucky and Ohio, 2024

Characteristic	Contact group, no. (column %)			
	Health care worker contacts	Household contacts	Other community contacts*	Total contacts
No. of potential contacts (row %)	645 (88)	7 (1)	84 (11)	**736 (100)**
Underwent risk assessment	645 (100)	7 (100)	57 (68)	**709 (96)**
PEP recommended^†^	53 (8)	7 (100)	0 (—)	**60 (8)**
Did not receive PEP^§^	1 (2)	3 (43)	0 (—)	**4 (7)**
Received partial PEP	5 (9)	1 (14)	NA	**6 (10)**
Completed PEP^¶^	47 (89)	3 (43)	NA	**50 (83)**

**Abbreviations**: NA = not applicable; PEP = postexposure prophylaxis.

* Includes close contacts in the workplace and classroom (i.e., coworkers, classmates, and instructors) and other community members.

^†^ Among those who underwent risk assessment.

^§^ Four persons who were recommended to receive PEP (one health care worker and three household contacts) did not complete PEP or receive partial PEP, despite multiple calls from local health department staff members.

**^¶^** Received at least 1 PEP dose but did not complete the vaccination series.

**Community contacts.** Overall, 91 household and community contacts were identified. The patient’s partner, roommates, and family members were contacted, and their exposure risk assessed. His employer provided a list of coworkers on his shift during his infectious period. Public health officials conducted outreach through email, telephone calls, text messages, and multilingual letters distributed at work and mailed to homes. They also contacted classmates and instructors from English classes the patient attended and a nurse who vaccinated him during his infectious period.

The patient traveled to New York for 3 days early in his infectious period. The New York State Department of Health assessed exposure risk among three relatives with whom he stayed and determined that all three had potentially been exposed to saliva. The patient traveled by plane, initially raising concern for exposure of other travelers. However, he was not exhibiting hypersalivation or agitation at the time, and the risk for passenger exposure to infectious fluids (e.g., saliva) during the short flights was deemed minimal. Therefore, contact tracing of others on the planes was not pursued.

### Recommendations for and Administration of PEP

Among 736 contacts identified in Kentucky, Ohio, and New York, 709 (96%) completed a risk assessment, 60 (8%) of whom were considered exposed through contact with saliva and recommended to receive PEP. These included 53 of 645 (8%) health care workers, all seven household contacts, and none of 57 other community contacts (Table). Local public health departments coordinated with the hospitals to ensure that rabies PEP administration aligned with Advisory Committee on Immunization Practices guidelines (*5*). Recommended PEP consisted of a single dose of human rabies immune globulin and 1 dose of rabies vaccine at the time of the first medical visit, followed by an additional vaccine dose on days 3, 7, and 14 after the first dose. Occupational health staff members at each hospital coordinated PEP administration for their respective employees, and community contacts’ PEP was monitored by local health department staff members. Among all 60 persons recommended to receive PEP, 50 (83%) completed the vaccination series; six persons received at least 1 dose of vaccine but did not complete the series. Public health staff members reviewed the telephone interview statements of each health care contact who was recommended to receive PEP to determine the circumstances of their exposure. Among 49 of 53 (92%) exposed health care workers, recommendations to receive PEP might have been avoided through adherence to standard precautions. In the remaining four cases, enhanced precautions would have been required because of the nature of the patient contact.

## Discussion

Human-to-human transmission of rabies has only been confirmed through organ or tissue donation. Although rabies transmission from patients to health care workers is theoretically possible, it has not been documented. However, because infected humans shed virus in saliva, these persons should be considered potentially infectious to others through exposure to infectious tissue or body fluids. In this case, the prolonged hospitalization and delayed consideration of rabies as a diagnosis increased the period during which health care workers could have been exposed to infectious material. Because rabies is nearly universally fatal after symptom onset, prevention is critical. This case represents one of the largest health care–associated rabies exposure investigations in recent U.S. history and suggests how adherence to recommended infection control precautions, including use of PPE, along with early public health consultation, might reduce the unnecessary administration of PEP.

While caring for this patient, health care workers had extensive contact with his saliva. In a health care setting, exposure to rabies virus could occur through contact between a patient’s saliva and a health care worker’s eye, mouth, or broken skin. Despite this, only 8% of those assessed were recommended to receive PEP. Standardized risk assessment can help direct PEP recommendations to persons most likely to be at risk and reassure those without exposure, minimizing possible adverse effects and cost of PEP by reducing unnecessary administration.

Most exposures in this investigation were health care associated (53 of 60; 88%). Standard infection control precautions are recommended when caring for all patients, including those with suspected rabies (*6*,*7*). Use of gloves, gowns, masks, and eye protection can protect against body fluid exposure, particularly during intubation and suctioning. Although standard precautions should be used for all patient care, delayed diagnosis of rabies in this case and health care workers’ lack of awareness of the risk for rabies transmission might have contributed to some health care workers’ failure to use recommended precautions.

Human rabies is rare in the United States, and most U.S.-based clinicians have never encountered a case (*8*). Rabies diagnosis might therefore be delayed or missed because of clinician unfamiliarity or hesitancy to consult with public health departments. Although rabies was considered early in this patient’s clinical course, testing was deferred while more common and easily tested diagnoses were assessed and ruled out. The typical rabies incubation period is approximately 3 weeks–3 months, although incubation periods of <1 week and >1 year have been reported (*9*). The long incubation period in this case (≥7 months) reduced the clinical suspicion for rabies. Although human rabies is rare, the virus remains enzootic in U.S. wildlife and is reported in mammals from all states except Hawaii. State health departments often have staff members who are experienced with rabies testing protocols and should be consulted promptly when rabies is suspected. Immediate public health consultation when rabies is being considered can prevent diagnostic delays and minimize exposures.

### Implications for Public Health Practice

This patient had recently arrived in the United States from one of the countries with the highest risk for rabies in the Western Hemisphere and experienced classic rabies signs and symptoms. This case underscores the value of early public health consultation when a diagnosis of rabies is considered. The case also highlights the importance of adhering to standard precautions during all patient care activities and the use of standardized risk assessments to ensure timely and effective response efforts.

## Footnotes

* Nomenclature for Caribbean rabies virus variants recently described in Frontiers|UsingmolecularapproachestodeterminerabiesdiversityinHaitiandDominicanRepublic.

† 45 C.F.R. part 46, 21 C.F.R. part 56; 42 U.S.C. Sect. 241(d); 5 U.S.C. Sect. 552a; 44 U.S.C. Sect. 3501 et seq.

## References

[1] Wallace R, Etheart M, Ludder F, The health impact of rabies in Haiti and recent developments on the path toward elimination, 2010-2015. Am J Trop Med Hyg 2017;97(Suppl):76–83. 10.4269/ajtmh.16-0647 29064363 PMC5676638

[2] Gigante CM, Dettinger L, Powell JW, Multi-site evaluation of the LN34 pan-lyssavirus real-time RT-PCR assay for post-mortem rabies diagnostics. PLoS One 2018;13:e0197074. 10.1371/journal.pone.0197074 29768505 PMC5955534

[3] Council for State and Territorial Epidemiologists. Revision of the surveillance case definition for human rabies. Atlanta, GA: Council for State and Territorial Epidemiologists; 2011. https://cdn.ymaws.com/www.cste.org/resource/resmgr/PS/10-ID-16.pdf

[4] Fooks AR, Jackson AC, eds. Rabies: scientific basis of the disease and its management. 4th ed. Cambridge, MA: Academic Press; 2020.

[5] Rupprecht CE, Briggs D, Brown CM, ; CDC. Use of a reduced (4-dose) vaccine schedule for postexposure prophylaxis to prevent human rabies: recommendations of the advisory committee on immunization practices. MMWR Recomm Rep 2010;59(No. RR-2):1–9. 20300058

[6] Manning SE, Rupprecht CE, Fishbein D, ; Advisory Committee on Immunization Practices CDC. Human rabies prevention—United States, 2008: recommendations of the Advisory Committee on Immunization Practices. MMWR Recomm Rep 2008;57(RR-3):1–28. 18496505

[7] Garner JS; The Hospital Infection Control Practices Advisory Committee. Guideline for isolation precautions in hospitals. Infect Control Hosp Epidemiol 1996;17:53–80. 10.1086/647190 8789689

[8] Ma X, Boutelle C, Bonaparte S, Rabies surveillance in the United States during 2022. J Am Vet Med Assoc 2024;262:1518–25. 10.2460/javma.24.05.0354 39059444

[9] Wilson PJ, Rohde RE, Oertli EH, Willoughby RE, eds. Rabies: clinical considerations and exposure evaluations. 1st ed. New York, NY: Elsevier; 2020.

